# The clinical learning environment, supervision and future intention to work as a nurse in nursing students: a cross-sectional and descriptive study

**DOI:** 10.1186/s12909-022-03609-y

**Published:** 2022-07-15

**Authors:** Juxia Zhang, Linda Shields, Bin Ma, Yuhuan Yin, Jiancheng Wang, Rong Zhang, Xueke Hui

**Affiliations:** 1 Educational Department, Gansu Provincial Hospital, Lanzhou, Gansu China; 2 School of Nursing, Midwifery and Indigenous Health, Charles Sturt University, Bathurst, New South Wales Australia; 3 Evidence-based Medicine, Lanzhou University, Lanzhou, Gansu China; 4 School of Nursing, Gansu University of Chinese Medicine, Lanzhou, Gansu China; 5 Geriatrics Department, Gansu Provincial Hospital, Lanzhou, Gansu China; 6 Lanzhou Medical and Medical insurance Supervision service Guidance Center, Lanzhou, Gansu China

**Keywords:** Nursing students, CLES+T, Intention

## Abstract

**Background:**

Clinical practice is a core component of nurse education. It is believed that nursing students’ clinical placement experiences can affect their learning outcomes, satisfaction, as well as influence their choice of future career. To examine nursing students’ perception of clinical learning environment and mentoring in hospital where they perform their clinical placement and the connection of these factor with intention to work as a nurse once graduated.

**Methods:**

Nursing students enrolled in clinical practice at least 6 months in hospitals in China were surveyed between January–March 2021. Percentages, frequencies, mean, standard deviation, t-test, ANOVA, and regression analysis were used to analyse the data.

**Results:**

Of the five scales in the CLES+T, ‘Leadership style of the ward manager’ scored the highest mean while ‘Pedagogical atmosphere at the ward’ scored the lowest. Nursing students with lower educational level, those supervised by fixed preceptor, and those intent to be a nurse in the future were significantly more satisfied with the CLES+T. Most of the nursing students are intent to work as a nurse in the future. CLES+T total scores and sub-dimensions (Premises of nursing on the ward) have significantly effectiveness on the intention to be a nurse in the future.

**Conclusions:**

Given the significant correlation of between learning environments and nursing students intention to be a nurse in the future, ward managers need to build a good clinical teaching atmosphere and promote opportunities for theoretical and practical connections among students through effective feedback mechanisms, which can enable students to experience a better clinical learning environment and meaningful experiences to build their professional roles and competencies, thus helping to enhance students’ willingness to pursue nursing careers in the future.

## Background

Nursing education consists of theory and practice. Clinical learning forms half of the educational experience of students in nursing education [1]. The theoretical part, conducted in classrooms and labs through lectures, case studies, and directive discussions, provide students with opportunities to develop their knowledge, skills, attitudes, and values taught. As complementary, clinical practice assists students in developing clinical knowledge and skills [2], integrating theory into practice [3], get opportunity to gain an insight into the real nurse’s roles [4] and meanwhile expanding their expectations of their future careers [5].

The clinical learning environment (CLE) is the interactive network of forces within the clinical setting that influence the students’ clinical learning outcomes [6], and it also has an impact on students’ internship preparation and satisfaction with the nursing profession [7]. In this new context, students learn how to apply practical skills, also interact with patients and other skills that acquired in the theoretical courses, in addition, considering nurses’ obligation to continue their professional development, their experience regarding CLE and mentoring models have an impact on their decisions and motivation as regards further workplace [7]. Unlike theory course where student’s learning activities are structured, students in clinical settings are often exposed to unplanned events, such as dealing with challenging patients and their families, poor supervision [8], lack of correspondence between the length of internship courses and the specified objectives [9], all which may cause students experience high levels of stress and anxiety [10] thus may influence their well beings, as well as choice of future career [8]. Thus, high quality and effective CLE is required to ensure nursing students be able to advance their competences and increase confidence as independent nursing professionals [11].

Considering the significance of clinical placement in nursing education, monitoring the CLE and to identify those elements which need to be addressed and improved are of great valued. A growing body of research has documented an association between the quality of CLE and nursing students satisfaction and well -beings. LevettJones et al. suggest that students’ sense of belonging in a clinical placement improves confidence and motivation in learning [12]. Evidence showed that mentor’s support is critical to the professional development of nursing students in clinical practice, and positive experiences of mentor can enhance students’ motivation to continue in the nursing profession [13]. Negative experiences of the learning environment are also evident such as dislike of one’s own department [14], and impoverished learning environments where staffs’ negative attitudes towards working with elderly pervaded the learning environment [15]. Pedagogical atmosphere characterized by respect, acceptance and opportunities for learning with the mentor and clinical teacher alike have a stake in making clinical learning successful and reliable [16]. Satisfied clinical learning environment and supervision would support the development of students’ clinical competence and have a significant effect on the outcomes of students’ experiences [17].

The educational importance of learning environments is reflected in the numerous tools developed to measure them [18]. The Clinical Learning Environment, Supervision and Teacher Scale (CLES+T) is one of them which developed in 2008 [19], aiming at multidimensional evaluation of nursing students’ perceptions towards clinical placement and for measuring the quality of clinical education in hospital units. In addition, having been translated into more than 27 languages, such as in Sweden [20]. Germany [21], Italy [22], Ghana [23] and Spanish [24], Dutch [25], Greek [16], Turkish [26], Korea [27], the scale is now used in over 40 countries [28]. Current studies in a variety of countries considered that CLES+T, which includes five basic elements of clinical learning such as supervision and/or mentorship, role of the nurse teacher, a learning-conducive atmosphere on the ward, nursing care provided on the ward, and the leadership style of the ward manager, can be a useful instrument to assess those elements of clinical learning environment at the international level [19].

China, similar to other countries, is experiencing nursing shortages. It is estimated that the nurses’ shortage was 3,46,000 in China in 2013 [29]. Consequently, it is crucial to have more students to consider nursing as a career, and one way to achieve this is to improve nursing students’ learning experiences during clinical practice period. Educational councils also consider medical students’ well-being as part of strategies to improve the quality of education and health care [30]. Nursing education in China comprises comprises three levels: Diploma; Advanced Diploma and Baccalaureate Degree. Despite the differences in nursing qualification levels, nursing graduates are required to take supervised experience in hospital setting as an irreplaceable component of nursing education [31]. These clinical placement is divided into intra lectures (students are placed for 2 day each week whiles they continue with their lectures and academic activities. This comprise of two to 4 weeks block) and final year (when students are finish their theory lectures and examination. This comprise of 8 to 12 months). In the final year clinical placement, students were allocated to different wards, and spent 40 hours per week in the clinical area with preceptor. The clinical preceptors sign off the clinical assessment of the students in each ward. The mean assessment of each ward constitutes the mark a student will score in his or her final clinical placement. Therefore, the need to improve clinical nursing education is an important aspect of training of students. Understanding nursing students’ assessments of their own well-being in clinical workplace may enable the development of direct strategies to improve outcomes of clinical learning.

Although studies provide strong empirical evidence of the importance of good clinical leaning environments and nursing students well-beings in Western countries, few studies have focused on the effects of CLE on students outcomes in China. In 2015, the reliability and validity of Chinese version of CLES+T was tested by Wang et al. and found this instrument is suitable for clinical use in Chinese culture with the Cronbach’s alpha was 0.945 [32]. However, awareness of CLES+T’s importance among the Chinese nursing students is quite limited. The limited studies cannot give a clear understanding about nursing students clinical learning environments in China or their effects on students further career chooses. As all of the nursing students required attending clinical placement, it is critical to examine the perception of students in such environment. The aims of this study were to (1) analyse nursing students’ perception of the clinical learning environment and supervision, and to identify the factors that affect these. (2) analyze the association between clinical learning environment and intention to stay in those hospitals once graduated.

## Methods

### Study design

This was a cross-sectional observational study among a convenience sample of nursing students participating in clinical placement in China. The study was reported in accordance with the STrengthening the Reporting of OBservational studies in Epidemiology (STROBE) checklist [33]. This study was approved by Gansu Provincial Hospital’s Ethic Commission (2018–106). All methods were carried out in accordance with the Declaration of Helsinki. Informed consent was obtained from all subjects and/or their legal guardian(s). We handled survey data confidentially and maintained anonymity of respondents throughout the study.

### Participants and setting

The sample size should be 5–10 times or 10–20 times of the number of questionnaire items. In order to ensure the reliability of the study, the preliminary sample size was estimated to be 340–680. Considering that invalid questionnaires may occur due to lack of information and filling errors during the questionnaire collection process, the sample size was relatively ideal to be 374–748, the final sample size is 660 [34].

A convenience sample was recruited from 6 tertiary hospitals in China, of each contains above 1000 patient beds and about 200 nursing students be enrolled for clinical practices per year. In China, the nurses are graded into four levels: Nurse 1 (1–2 years), Nurse 2 (3–5 years), Nurse 3 (6–10 years), Nurse 4 (10–20 years). Only the nurses above N1 have the qualifications to supervise students. There are two types of supervision model: fixed model, which means “preceptor at one to one basis,” and not fixed model, where the preceptor could change from 1 shift to another. The student nurses were allocated in various nursing departments. For inclusion, the students should: (1) enrolled in the clinical practices at least 6 month, (2) voluntary to attend the survey.

### Research instrument

This CLES+T scale consists of 34 items and was divided into five sub-dimensions: ‘Role of nurse teacher’, ‘Supervisory relationship’, ‘Pedagogical atmosphere at the ward’, ‘Leadership style of the ward manager’ and ‘Premises of nursing on the ward’ [2]. Each item uses a 5-point Likert scale. The scores were as follows: 1 = fully disagree, 2 = disagree to some extent, 3 = neither agree nor disagree, 4 = agree to some extent and 5 = fully agree. We added questions on programme of study and level where higher scores indicate more agreement with the statements. The internal consistency of the the instrument and each dimension was estimated with Cronbach’s alpha coefficients.

### Data collection

Approximately 6 nursing managers from the sampling hospitals were selected to be the ‘original deliverers’ of the survey. The original deliverers were alumni who maintained friendly contact with the researchers and had nursing education positions in various hospitals. The investigators converted the questionnaire into the Questionnaire Star platform, and then forwarded it on the WeChat platform which is the most popular communication and social platform in China. All items are required to be filled in, and the same IP address can only be answered once. Prior to the formal online survey, we provided comprehensive survey training to these initial contacts, and commissioned them to recruit 20 nursing students to answer the questionnaire during the same period. Then, a Wechat account link to our questionnaire was sent by mobile phone to the original deliverers who were asked to finish the data collection within 2 days. The original deliverers choose the time to fill in the questionnaire according to their situation, and then post the Wechat account to the student groups. The amount of data collected was monitored in real time on the website’s management platform. Through the preliminary survey of 20 students, we counted the average time to complete the questionnaire, and the shortest time was more than 4 minutes. Therefore, the quality of the questionnaire completed within 3 minutes was difficult to guarantee, so it was excluded. In addition, questionnaires that miss more than two-thirds of the total number of questions are considered invalid and therefore should be excluded [34]. The questionnaires were being distributed from January to March 2021.

### Statistical analysis

SPSS 19.0 was used for statistical analyses. Frequencies and percentages was used to examine demographic data. An overall mean score of the questionnaire was calculated for each student by calculating the mean score of all questions. Scores on the five sub-dimensions were also calculated for each student using scores of the questions that make up those dimensions. The association between demographic characteristics and clinical placement experience and mean scores was determined using t-test or ANOVA as appropriate. We determined correlation of the overall mean score on CLES+T and demographic characteristics (binary/categorical) using the linear regression. Logistic regression was used to evaluate effect of the CLES+T on participants’ attitudes to employment choices.

## Results

### Reliability and validity of the questionnaire

The results showed that CVI = 0.916, the range of CVI of each item was 0.866 ~ 1.00, CVR = 0.858. The Pearson correlation coefficient between each factor was 0.437-0.485, and the correlation coefficient between each factor and the total score of the scale was 0.662- 0.791.

The total Cronbach’s coefficient of the questionnaire was 0.931. Cronbach’s instrument coefficients of each factor were 0.916, 0.893, 0.908, 0.887 and 0.869, respectively. The MIIC value of the questionnaire was 0.295, and the MIIC value of each factor was 0.653, 0.618, 0.667, 0.659 and 0.638.

### Sociodemographic characteristics of nursing students

A total of 660 nursing students completed the questionnaire, and this equals a response rate of 82.5%. Table 1 shows demographic characteristics. The participants were between 19 and 25 years old (mean 22). The majority (91.2%) were female and most (61.7%) are having an advanced diploma, 75.6% came from rural area. More than half are supervised by staff according to different shifts. Most (65.4%) are supervised by nurse in N2, and 77.6% nursing students intended to be a nurse in the future (Table 1).

**Table 1:** Sociodemographic characteristics and CLES+T scores for example (*N* = 660)

Characteristics	Frequency N(%)	CLES + T score mean (SD)	F/t	*P*
Gender			1.58^b^	0.11
Male	58 (8.8)	135.00 ± 25.19		
Female	602 (91.2)	129.09 ± 20.97		
Home location			0.89^b^	0.37
Rural area	499 (75.6)	129.07 ± 20.75		
Urban area	161(24.4)	131.27 ± 23.35		
Educational level			1.44^b^	0.01*
Bachelor’ s degree	253 (38.3)	121.51 ± 22.42		
College degree	407 (61.7)	134.26 ± 19.45		
Current practice ward			0.79^a^	0.88
Internal medicine	135 (20.4)	129.76 ± 21.146		
Surgery	223 (33.8)	130.63 ± 23.95		
O&G	90 (13.6)	133.77 ± 20.13		
Pediatric	29 (4.5)	119.57 ± 22.17		
Operating theater	92 (13.9)	129.15 ± 15.10		
Acute &Emergency	86 (13.1)	124.38 ± 22.84		
ICU	5 (0.8)	139.44 ± 17.13		
Model of supervisor			1.62^b^	0.00*
fixed	271 (41.5)	136.54 ± 19.36		
Not fixed	386 (58.5)	124.99 ± 21.29		
Title of preceptor			1.15^a^	0.19
Nurse 1	73 (11.0)	125.80 ± 22.87		
Nurse 2	431 (65.4)	131.15 ± 21.31		
Nurse 3	156 (23.6)	127.06 ± 20.82		
Intention to work as a nurse in the future			5.27^b^	0.00*
Yes	512 (77.6)	131.65 ± 20.49		
No	148 (22.4)	115.27 ± 31.12		

^a^ equal to F value; ^b^ equal to t value;

*P < 0.05

### CLES+t

The means and medians for total and the five subscales were shown in Table 2. Overall, the students evaluated the clinical learning environment positively. The mean values (range from 1 to 5) for the sub-dimensions varied between 3.77 for ‘*Pedagogical atmosphere at the ward’* to 4.02 for *‘Leadership style of the ward manager’* (Table 2).

**Table 2:** Association between CLES+T scales/subscales and the item on “Intention to be a nurse in the future” in terms of logistic regression

Scales/subscales	$\overline{x}$±SD	B	S.E.	Wald	*P*	OR	95%CI	
							Lower	Upper
Total scores	129.61 ± 21.41 (Range: 34–170)	− 0.06	0.08	61.09	0.00*	4.83	0.92	0.95
**Dimensions of CLES + T**								
Supervisory relationship	3.99 ± 0.708	−0.12	0.41	0.02	0.72	0.88	0.89	1.08
Pedagogical atmosphere at the ward	3.77 ± 0.766	1.75	0.40	17.09	0.00*	5.67	0.08	0.95
Role of nurse teacher	3.98 ± 0.623	0.34	0.60	0.23	0.68	1.38	0.88	1.17
Leadership style of the ward manager	4.02 ± 0.649	0.53	0.41	1.48	0.29	1.72	0.66	1.18
Premises of nursing on the ward	3.99 ± 0.644	−1.60	0.52	9.82	0.00*	0.20	0.60	1.28

*P < 0.05; OR, odds ratio; *CI* Confidence interval;

Mean scores for single items varied from 3.41 to 4.24. The three highest score was given to the item *The ward nursing philosophy was clearly defined* (4.24 ± 0.620), *The ward manager was a team member* (4.19 ± 0.65), and *There was a mutual interaction in the supervisory relationship* (4.16 ± 0.74). The three lowest score was given to items measuring Pedagogical atmosphere on the ward: *I felt comfortable going to the ward at the start of my shift* (3.41 ± 1.07), *The staff learned to know the student by their personal name,* (3.53 ± 1.13), and *During staff meetings (*e.g. *before shifts) I felt comfortable taking part in the discussions* (3.57 ± 1.10) (Table 3).

**Table 3:** Single items with the 3 highest and lowest mean score

Scores	Scales/subscales	$\overline{x}$±SD
The 3 highest score	The ward nursing philosophy was clearly defined	4.24 ± 0.62
	The ward manager was a team member	4.19 ± 0.65
	There was a mutual interaction in the supervisory relationship	4.16 ± 0.74
The 3 lowest score	I felt comfortable going to the ward at the start of my shift	3.41 ± 1.07
	The staff learned to know the student by their personal name	3.53 ± 1.13
	During staff meetings (e.g. before shifts) I felt comfortable taking part in the discussions	3.57 ± 1.10

Analysis using the t-test/ one-way ANOVA examined differences in CLES+T scale with different students characteristics (Table 1). Further analysis compared differences in nurse outcomes among nurses with different characteristics. The findings showed that nursing students who are having advanced diploma experienced more positive clinical learning environment than those having bachelor’s degree (t = 1.44, *p* = 0.01), students supervised by fixed supervisor are more satisfied with clinical learning environment than others (t = 1.62, *p* = 0.00), and those who had the intention to work as a nurse in the future were more satisfied with clinical learning environment than whose not (t = 5.27, *p* = 0.00).

### Effects of students characteristics on clinical learning environments

The logistic regression analysis showed that educational level, model of supervisor and like to be a nurse in the future could significantly predict satisfaction with clinical learning environments (Table 4).

**Table 4:** Correlation between general characteristics and total CLES+T scores

Characteristics	Unstandardized Coefficients		Standardized Coefficients	t	*P*	95% CI	
	B	SE	Beta			Lower	Upper
Gender	6.85	3.50	0.08	1.95	0.06	−0.02	13.73
Educational level	−11.74	2.28	−0.25	−5.14	0.00*	−16.22	−7.25
The class of included hospital	5.02	2.81	0.08	1.78	0.07	−0.51	10.55
Home location	2.91	2.28	0.05	1.27	0.20	−1.58	7.40
Model of supervisor	9.67	2.05	0.21	4.71	0.00*	5.64	13.70
Like to be a nurse in the future	12.07	3.10	0.17	3.88	0.00*	5.96	18.18

95% CI, 95% confidence interval for B; SE, standard error; *P < 0.05

### Effects of clinical learning environments on students intention to be a nurse

Most of the nursing students (77.6%) were intent to work as a nurse in the future (Table 1). Further analysis association between in intent among students and CLES+T scales/subscales. The negative skewness values for total scores (− 0.06) and sub-dimensions (Premises of nursing on the ward, − 1.60) lean in the direction of positive values of the item intention to be a nurse in the future (Table 2).

## Discussion

This study provides a view on Chinese nursing students’ clinical learning environment and supervision in the hospital where they undertook clinical placements. On the whole the students perceived a favourable clinical learning environment and supervision, recording high levels of intention to be a nurse in the future, which may also be related to the fact that more than 60% of the students in our study have college degree. In China, there are about 800,000 nursing graduates each year, including about 60,000 to 80,000 undergraduates and 350,000 college students [35]. Therefore, compared with undergraduate students, the proportion of college students is larger. Our study showed that college degree students have higher CLES+T scores than undergraduates, and those with higher CLES+T scores were more likely to be interested in a future career in nursing. Evidences showed that nurses with university degrees offer better nursing care when compared to nurses with other qualifications [5]. Nurses with college degree have higher level of reasoning, think critically and make sound clinical judgements when executing patient care [36]. With higher academic qualifications, undergraduates are more likely to have psychological superiority, which may cause dissatisfaction of others and increase contradictions and conflicts with others. Currently, the majority of clinical nurses in China are still those with bachelor’s degree or less. Furthermore, semi-structured qualitative interviews could be a feature of future studies examining causal links between education background and clinical learning environment and supervision of nursing students.

Our findings demonstrated that pedagogical atmosphere at the ward is the factor influencing student nurses’ motivation to choose nursing as a career. Students need a clinical learning environment and atmosphere that is supported, respected and encouraged, which is essential for the mastery of clinical practice skills [16]. A good clinical learning environment is created through an inspiring learning atmosphere, student orientation to the work environment, and a positive interpersonal relationship between students and tutors [37]. The positive learning atmosphere allows students to have more positive relationships with other team members, to truly feel involved in ward activities, and to be more motivated to explore new skills in clinical practice [38]. However, the leadership style of ward managers remains a key element of experiential learning in clinical Settings [39]. However, a good learning environment is characterized by democratic leadership style, if ward managers aware of students’ physical and emotional needs, and encourage them to participate in all kinds of promoting learning experience, which will help to stimulate students’ interest in clinical practice [40]. Compared with other studies where the CLES+T instrument has been used, the students’ mean values for the sub-dimensions (between 3.77 to 4.02) are similar with studies evaluated students in nursing home [41] and hospital settings [23]. One prominent feature of the present study is that the dimension ‘Leadership style of the ward manager’ has the highest mean score. Students in our study evaluated the leadership style more in line with hospital setting [23]. However, it is contrast with the study by Carlson et al.which calculated the sub-dimension with lowest score in nursing home. Evidence showed that leadership style was positively associated with job satisfaction, staff retention, costs, quality of care [42], quality of work life, coping style [43] and work environment [44], thus the clinical leadership styles of managers can be crucial in the ward which has a significant role in furthering employees’ sense of self-efficiency, which in turn helps promote job satisfaction. Multifactorial Leadership Model states that employees tend to be attracted by leaders who show an enthusiastic and optimistic nature and who know how to make long-term plans [44]. It is possible that the attractive clinical leadership style provide a safe learning environment when students know what is expected of them. We suggest that there is a need both of further empirical studies on the role of clinical leaders in undergraduate nursing education and of studies comparing different leadership styles.

Feedback on nursing students’ clinical performance and satisfaction is critical to their effective learning in clinical practice [45]. However, this is not doing well in our study as most preceptor do not know students’ name, and students do not feel good when going to the ward or taking part in the discussions. During clinical practices, it is one of the preceptor’s major responsibilities to provide students more opportunities to participate ward activities, thereby creating higher levels of enthusiasm and motivation for learning while at the same time increasing their self-confidence [46]. This contributes to the improvement of nursing students’ satisfaction with ward nursing work, which is another factor influencing students’ career intention. Our results showed that model of supervisor was an independent influencing factors of CLES+T scores, students with fixed teachers had higher CLES+T scores. A student is either assigned to an experienced clinical nurse who will guide the student throughout the placement on a one-to-one basis [47]. Although less students received supervision from one-to-one basis during their clinical placement, the study found that students in this model (had the same preceptor all the time) were more satisfied with their clinical learning environment than those who had different preceptors each day. These findings are consistent with other studies [5, 48], which indicated that students with the same preceptor throughout were more positive concerning the supervisory relationship and the pedagogical atmosphere. Papastavrou et al. Found that the one-to-one relationship between students and tutors helps students to increase their role socialization, clinical competence, self-confidence, and critical thinking [49]. Meanwhile, Myall et al. found that most students preferred to assign a fixed teacher because the teacher provided them with feedback and learning opportunities [50]. In addition, the one-to-one tutorial system provides students with opportunities to develop self-confidence, which is conducive to the improvement and development of students’ psychomotor skills, which has a great impact on the choice of nursing students’ career [49, 50].

Interestingly, we found that students supervised by N2 nurses had higher CLES+T scores. The proportion of primary nurses in hospitals is increasing. Since they do not have sufficient clinical experience, it is reasonable to assume that they may not be able to properly supervise and instruct students [51]. However, Phuma-Ngaiyaye et al.’s study showed that N2 nurses can provide students with more teaching experience to facilitate their learning [52]. Compared with N3 nurses, N2 nurses had more opportunities to contact with students, so that students can not only feel good learning atmosphere, as well as to improve the students’ ability of clinical practice [53]. Therefore, it’s not surprising that students under the guidance of an N2 nurse showed higher CLES+T scores. Since the clinical role of the nurse teacher has changed from a clinical skilled practitioner to a liaison person working between educational and health care provider organisations, we suggest that training institutions or teaching hospitals should be accredited using a set of minimum accreditation standards that reflect nurses’ clinical teaching competency. Only accredited nurse educators would then be used for the clinical teaching of students [54]. Meanwhile, building a dedicated work team is an effective strategy to improve the clinical learning experience of nursing students, which can enable students to experience a better clinical learning environment and meaningful experiences to build their professional roles and competencies [55].

### Limitations

This cross-section study focused on the learning challenges of a group of nursing students in teaching hospital of China. Therefore, the generalization of the findings should be done with caution and it is necessary to conduct further studies on this in different areas to find the various factors effective on students’ learning environment, and subsequently have a better understanding in teaching nursing students in their clinical practice.

## Conclusions

We used a quantitative study to examine student nurses’ roles in learning in clinical placements in China. It is expected that students play an active role in the learning and training process during clinical placements. Support and supervision can promote a clinical learning atmosphere and interaction with peers, thereby enhancing the nursing student’s preference for the nursing profession. Ward managers need to build a good clinical teaching atmosphere and promote opportunities for theoretical and practical connections among students through effective feedback mechanisms. Building a dedicated work team is an effective strategy to improve the clinical learning experience of nursing students, which can enable students to experience a better clinical learning environment and meaningful experiences to build their professional roles and competencies.

## Data Availability

The datasets used and/or analysed during the current study are in Chinese and are available from the corresponding author on reasonable request but will require translation to English.
